# The Geometry of Language: Understanding LLMs in Bioethics

**DOI:** 10.1007/s11673-025-10480-1

**Published:** 2025-09-11

**Authors:** Aníbal M. Astobiza

**Affiliations:** Departamento de Filosofía I, Edificio de la Facultad de Psicología, Universidad de Granada, Campus de la Cartuja, 18011 Granada, Spain

**Keywords:** Large Language Models (LLMs), Bioethics, Linguistic ambiguity, Semantic topology, Colexification

## Abstract

In this article, I explored the application of large language models (LLMs) in analysing linguistic colexification and ambiguity within bioethical scenarios. By employing word embeddings derived from LLMs, I constructed semantic distance matrices that provide insight into the relationships between key terms in bioethical vignettes. These matrices were used to quantify and visualize the degree of linguistic ambiguity and specificity across different versions of each vignette—those with high colexification (ambiguous language) and those with low colexification (specific language). The approach taken involves encoding words according to their semantic adjacency and representing these relationships geometrically through distance matrices. The resulting matrices reflect the nuanced differences in how concepts are related within bioethical contexts, offering a quantitative method for analysing language use. The study demonstrates that LLMs, by facilitating geometric representations of language, can enhance our understanding of complex ethical dilemmas by systematically addressing linguistic ambiguity. Ultimately, this research contributes to the field of bioethics by providing a computational approach to improving clarity in ethical communication, highlighting the potential of LLMs to inform both ethical decision-making and discourse analysis. LLMs, while not capable of performing speech acts in the full philosophical sense—as human beings do—still serve as powerful tools to analyse and understand bioethical language. This distinction—between performing speech acts and analysing their linguistic features—highlights the unique contribution of LLMs as analytical tools rather than ethical agents.

**Supplementary Information:**

The online version contains supplementary material available at 10.1007/s11673-025-10480-1.

## Introduction

The intersection of language and artificial intelligence (AI) presents a compelling opportunity to reconsider how ethical discourse^1^ can be analysed and understood. As technology continues to evolve, the potential for Large Language Models (LLMs) to assist in ethical deliberation has grown significantly (Cohen 2023; McMillan 2023). However, despite their impressive linguistic capabilities, LLMs do not fully replicate human communicative acts such as speech acts in the classical sense described by pragmatics and the philosophy of language (Searle 1969; Tsohatzidis 2002). For instance, an LLM can produce a sentence like “I promise to deliver the results,” but it does not genuinely intend to carry out any action or understand the implications of a promise. Similarly, it can generate a scary story, but it cannot truly grasp the emotional impact it might have on a reader or intend to evoke fear. These limitations stem from the fact that LLMs are essentially complex algorithms trained on vast amounts of text data without anchor or semantic grounding in the world. They are not embodied. They only excel at pattern recognition and language manipulation, but they do not possess genuine beliefs, desires, or an understanding of the world in the way humans do. Therefore, while LLMs can mimic human language, they cannot truly engage in the nuanced and complex act of communication. Instead, they can support ethical inquiry by offering sophisticated ways to examine and quantify linguistic elements of ethical discourse, enabling researchers to better understand the structure (geometry) and dynamics of ethical conversations. In this study, I propose a novel approach to analysing bioethical texts by employing structured matrices and leveraging the capabilities of LLMs to aid ethicists in their work^2^. Ethical dilemmas often involve complex decision-making processes that are heavily reliant on precise language. Bioethical discussions, which range from end-of-life decisions to genetic testing and organ donation, are filled with nuanced concepts that must be articulated with clarity, even sometimes appealing to etimology and words historicity, to support informed decision-making (Zwart 2021). However, the language used in such discussions can often be ambiguous, leading to misunderstandings or ethical misinterpretations. In this context, colexification—the phenomenon where one word expresses multiple meanings—can contribute to ambiguity, particularly in high-stakes bioethical scenarios. This ambiguity complicates the process of ethical decision-making, as different interpretations can lead to divergent moral conclusions.^3^ In this work, I employ a methodology based on the conversion of bioethical vignettes^4^ into structured matrices that quantitatively represent semantic relationships between key terms. Specifically, I utilize matrices representing semantic distances between words within the text and use various visualizations—such as hierarchical clustering dendrograms—to elucidate patterns in language use. The vignettes analysed in this study consist of both High Colexification (HC) and Low Colexification (LC) versions of bioethical scenarios. The HC versions are characterized by general, ambiguous language, while the LC versions feature more precise and specific terminology. The hierarchical clustering dendrograms provide insights into the structure of relationships among words, helping identify how closely words are related in terms of meaning within a given context. The SVD analysis, on the other hand, offers a means to assess the dimensionality of variance within the semantic space of each vignette, helping to capture the complexity of the language used. Heatmaps are used to visualize the overall coherence of the text, highlighting patterns in semantic distance that indicate levels of specificity or ambiguity. Through these analyses, I have observed a recurring pattern across the vignettes: a reduction in semantic distance and a more coherent structure in LC versions, suggesting that specificity in language use leads to tighter clustering of semantic concepts. This phenomenon, which I term the “Lexical Clarity and Semantic Adjacency Principle” implies that the clarity of language directly affects the coherence and interpretability of ethical discourse. Specifically, more precise language reduces the semantic distance between key terms, resulting in a more structured and less ambiguous representation of the ethical scenario. The implications of these findings are twofold. First, they suggest a systematic approach to evaluating the clarity of ethical discourse through the use of semantic distance matrices and visual analysis. By transforming ethical texts into matrices and using LLMs to analyse these structures, ethicists can gain new insights into how language shapes ethical decision-making. This quantitative approach provides a novel lens through which to assess the coherence of ethical arguments and identify areas where greater specificity may be needed. Second, this study highlights the potential for LLMs to assist in ethical analysis, not by performing speech acts themselves, but by providing a powerful tool for the examination of ethical language. While LLMs may lack the intrinsic intentionality required for genuine speech acts—such as making promises or issuing commands—they excel in processing large volumes of text and identifying linguistic patterns. By leveraging these capabilities, LLMs can help ethicists understand and improve the structure of ethical discourse, ultimately supporting more informed and ethically sound decision-making processes. The exploration of the “Geometry of Language” through the lens of LLMs and structured matrices offers a unique contribution to the field of bioethics. It provides a methodology that can be systematically applied to any bioethical text or transcript, helping ethicists analyse the linguistic components of ethical discussions in a rigorous, quantitative manner. This approach not only deepens our understanding of how language influences ethical reasoning but also lays the groundwork for future research into the role of AI in supporting ethical inquiry and decision-making. The remainder of this article is structured as follows. In Section"Background: Language Models and Ethical Discourse", I provide a detailed overview of language models and their relevance to ethical discourse. Section"Methods: Structured Matrices for Bioethical Analysis"presents the methodology, including the construction of structured matrices from bioethical vignettes. In Section"Visual Analysis Techniques", I describe the visual analysis techniques used to examine the matrices, such as hierarchical clustering. Section"Lexical Clarity and Semantic Adjacency Principle"discusses the Lexical Clarity and Semantic Adjacency Principle derived from the analysis, while Section"Discussion: Bioethical Analysis and LLMs"explores the implications for ethical analysis using LLMs. Finally, Section"Future Directions"offers concluding remarks and directions for future research.

## Background: Language Models and Ethical Discourse

The rapid evolution of LLMs in recent years has led to both enthusiasm and scepticism regarding their application to complex domains, such as bioethics (Ferrario and Biller-Andorno 2024; Haltaufderheide and Ranisch 2024; Porsdam Mann et al. 2023). The debate centres not only around what these models can achieve (Chen et al. 2024) but also around their theoretical implications for a variety of fields such as education (Khan 2024), science (Acquaviva 2023), and finance (Gambacorta et al. 2024). This article will focus on the implication of LLMs for bioethics. In this section, I will review the development and role of LLMs in the analysis of language, drawing connections between their computational capabilities and the challenges of ethical discourse, while also contrasting these capabilities with traditional approaches in linguistics and bioethics. According to Steven Piantadosi’s outline (2023), LLMs represent a major departure from the generative linguistic models traditionally associated with Noam Chomsky’s approach, which emphasizes innate language capabilities and the autonomy of syntax. Instead, modern LLMs, such as GPT-3 and GPT-4, rely on deep learning architectures that are capable of acquiring linguistic competencies, including grammar, semantics, and even pragmatic elements, through exposure to large datasets of text. These models do not require explicit, pre-defined syntactic rules; rather, they infer rules and relationships directly from data, integrating syntax and semantics seamlessly within vector-based representations. The ability of LLMs to generate coherent discourses, understand contextual nuances, and simulate various styles of speech has been achieved through innovations like attention mechanisms and vast parameter spaces. For instance, as Piantadosi notes, LLMs have successfully integrated gradient computations (Piantadosi 2023, 18), allowing them to predict text by focusing on both local and nonlocal dependencies. These models do not require explicit, pre-defined syntactic rules; rather, they infer rules and relationships directly from data, integrating syntax and semantics seamlessly within vector-based representations (Piantadosi 2023). This capability is particularly important for bioethical discourse, where language often operates on multiple levels of meaning—embedding ethical principles, conveying factual information, and appealing to the emotions of stakeholders.

However, the application of LLMs to ethical discourse raises unique challenges. As explored in the Hastings Center Report by Amy Gutmann and James Wagner (2017), ethical decision-making, particularly in a public context, often requires democratic deliberation that incorporates diverse perspectives and values. Bioethics, therefore, is not simply a matter of understanding language at the syntactic or semantic level; it also involves pragmatic components, such as speech acts, promises, and moral appeals, which go beyond the direct capabilities of LLMs. Moreover, bioethics has traditionally been shaped by what Clapp et al. (2024) refer to as the “representational view” of language—a view that sees language primarily as a means of description. This view has influenced the paths taken by bioethical theories and interventions, often limiting the potential of linguistic engagement in ethical deliberation. Clapp et al. advocate for a “pragmatic view” of language that emphasizes the performative aspects of communication and how language actively shapes ethical practices. Through the application of the pragmatic view to bioethical decision-making settings, they show that many of the problems with existing methods stem from their adherence to the representational view. Clapp et al. (2024) contend that the pragmatic perspective provides a more dynamic understanding of how language can be utilized to actively interact with ethical issues. Despite the above mentioned limitations, LLMs can still play a significant role in supporting bioethical inquiry. While they may not engage in speech acts in the classical sense—lacking intentionality and genuine performative capacity—they are highly effective at processing and analysing large volumes of text to extract patterns, co-occurrences, and areas of ambiguity. By leveraging these capabilities, LLMs provide new avenues for analysing ethical discourse, which can help ethicists understand how language is being used to frame ethical questions, identify potential sources of misunderstanding, and ensure that key concepts are articulated clearly. The emphasis on democratic deliberation in bioethics, as highlighted by Gutmann and Wagner, underscores the need for tools that can facilitate meaningful engagement with diverse perspectives. Here, LLMs offer unique value: they can assist in summarizing discussions, highlighting points of agreement and contention, and even suggesting alternative phrasings to make ethical arguments more accessible to laypeople and experts alike. Through such applications, LLMs can help bridge the gap between complex ethical language and the public’s understanding, thereby enhancing the deliberative process (Fleck 2024). Here I intend to use LLMs to gain a systematic comprehension of ethical discourse by turning bioethical texts into structured matrices that quantify semantic links. Semantic distances between important terms are represented numerically in the matrices created in this study, and these can be seen by clustering methods. Through this approach, it becomes easier to see how ethical arguments are structured and how language may either make or obfuscate ethical issues. All things considered, the emergence of LLMs has created new avenues for the analysis and comprehension of ethical discourse. Although the complete range of human communication cannot be replicated by these models, especially when it comes to intentionality and performative components of speech acts, their computational capabilities provide important insights into the ethical language. LLMs can support ethicists in elucidating arguments, spotting ambiguities, and guaranteeing that ethical conversations are carried out with the highest accuracy and clarity by methodically examining the linguistic structure of ethical discourse. The approaches for this study, which included transforming bioethical vignettes into structured matrices and analysing the results to identify patterns in ethical language, will be covered in detail in the parts around methodology.

## Methods: Structured Matrices for Bioethical Analysis

In this section, I describe the methodological approach adopted for transforming bioethical discourse into structured matrices to enable a systematic, quantitative analysis. This approach helps reveal underlying linguistic structures and patterns, providing insights into how different levels of language precision affect ethical deliberation. The methodology involves constructing bioethical vignettes, converting them into semantic matrices, and using advanced visualization techniques to interpret the data.

### Bioethical Vignettes and Colexification

The core data source for this study is a set of bioethical vignettes,^5^ each addressing significant topics within the domain, such as end-of-life care, informed consent, and organ donation. Each vignette was prepared in two distinct versions: High Colexification (HC) and Low Colexification (LC). The HC versions use language that allows multiple meanings for some words, inherently leading to a broader, often ambiguous interpretation. On the other hand, the LC versions strive for linguistic specificity, minimizing the use of ambiguous terms to ensure a more precise, focused discussion. For vignettes and their various versions, please refer to the online supplementary material linked to this article.

The goal of creating both HC and LC versions was to investigate how language precision impacts the structure and interpretation of bioethical discourse. This distinction allowed us to directly assess the influence of colexification on ethical decision-making and the coherence of the discourse, focusing on how these different levels of linguistic specificity affected the relationships between key concepts. To analyse the linguistic content of the vignettes, I constructed semantic distance matrices using Word2Vec word embeddings. This approach allowed me to quantify the relationships between key terms within the vignettes, based on their semantic similarities or differences. Broadly, Word2Vec operates as a shallow neural network that learns distributed representations by predicting neighbouring words within a context window (Mikolov, et al. 2013). Through this process, words frequently co-occurring in similar linguistic environments converge in vector space, reflecting semantic affinity. In practical terms, each token is transformed into a numeric vector whose dimensions capture latent semantic features, thus enabling the calculation of similarities (or dissimilarities) among tokens. This methodology, widely used in modern NLP pipelines, allows for flexible modelling of lexical meaning in large corpora and stands as one of the standard approaches for vectorizing textual data in empirical studies.

### Matrix Construction and Semantic Distance

The transformation of bioethical vignettes into structured matrices involved the creation of semantic distance matrices that represent the relationships between words based on their meanings. This process was carried out using word embeddings derived from a pre-trained Word2Vec model (The TensorFlow Authors 2020). The following steps detail the construction of these matrices: A) Tokenization: Each vignette was tokenized into individual words, allowing for a breakdown of the language into discrete components. Each token represented a key term or phrase relevant to the bioethical context presented in the vignette. B) Word Embedding Generation: Using Word2Vec, each token was converted into a high-dimensional vector. Word2Vec is capable of capturing the contextual meaning of words by analysing large corpora of text. The resulting vectors represent semantic properties of the words and their relationships within the corpus used to train the model. C) Calculation of Semantic Distances: Once the word embeddings were generated, the next step involved calculating the semantic distance between each pair of words. This was accomplished using cosine similarity, a common metric used in natural language processing to measure the closeness between two vectors. Cosine similarity values were transformed into distance values (1 - cosine similarity) to construct a symmetric matrix representing the semantic relationships within each vignette. D) Matrix Representation: The resulting semantic distance matrices were formatted to reflect the relationships between all key terms within each vignette. For HC vignettes, the matrices tended to have greater distances between certain key terms, reflecting the ambiguity and variability in meaning caused by colexification. Conversely, LC matrices exhibited smaller semantic distances, indicating more direct and less ambiguous relationships between terms.

$$CosineSimilarity=\frac{A\cdot B}{\left|A\right|\left|B\right|}$$

Where:

A and B represent the vector embeddings of two words.

||A|| and ||B|| are the magnitudes of the vectors.

The cosine similarity values range from −1 to 1, where a value close to 1 indicates high semantic similarity, while a value closer to 0 or negative indicates low or opposite similarity. To construct the semantic distance matrices, I converted the cosine similarity scores into semantic distances. This was done using the formula:

$$\text{Distance}=1-\text{Cosine Similarity}$$

This transformation makes the interpretation of relationships more straightforward: a lower distance indicates a close semantic relationship, whereas a higher distance indicates greater dissimilarity. Using these distance values, I constructed matrices for each vignette version. The LLM-derived embeddings enabled a more accurate representation of the relationships between key terms, especially when dealing with highly ambiguous or context-sensitive language. The resulting matrices provide a systematic way to analyse linguistic ambiguity and colexification across different bioethical contexts. Let’s work with the two versions of Vignette 1 (“Informed Consent in Medical Procedures”) as an example, detailing the encoding for both the High Colexification (HC) version and the Low Colexification (LC) version.

Definition of the word vector:

- **HC Version**:
- Relevant words: [“treatment”, “improve”, “condition”, “options”, “clear”, “decide”, “plan”, “suggested”]
- These words have a degree of ambiguity, as each can refer to different aspects without much specificity.

**LC Version**:

- Relevant words: [“surgery”, “replacement”, “hip”, “osteoarthritis”, “risks”, “benefits”, “infection”, “clots”, “recovery”]
- These words are more specific and provide details that reduce the ambiguity of the dilemma

Assignment of numerical values:

- A matrix is created in which each row and column represents a word, and the value within the matrix represents the semantic distance between those two words. These values are computed using a combination of methods, including semantic embedding spaces to quantify similarity, co-occurrence frequency from relevant corpora, and manual assessment of conceptual proximity. The resulting distances reflect how closely related the meanings of the words are within the context of the vignette. In the context of the Word2Vec model used (Mikolov et al. 2013), the numerical values in the matrices are derived based on how Word2Vec represents words in a high-dimensional embedding space. Here’s a detailed explanation of how this works and how the values are assigned to tokens. Word2Vec is a neural network-based model that converts words into dense numerical vectors of fixed dimensions, often called word embeddings. I used the Google Collaboratory Notebook by The TensorFlow Authors (2020). Each word is represented as a vector of real numbers (e.g., in a 100-dimensional space). Words with similar meanings are placed close together in this high-dimensional space. These vectors capture semantic information—words that have similar meanings or that are used in similar contexts will have similar vectors. I trained the Word2Vec model on a corpus (in this case, the bioethical vignettes), the model learned to assign each word a specific vector based on its contextual usage across the corpus. For instance, if “treatment” often appears in similar contexts as “therapy” Word2Vec will learn to give these two words vectors that are close to each other in the embedding space.

The complete set of cosine similarity matrices, representing both High Colexification (HC) and Low Colexification (LC) versions of the bioethical vignettes, is included within the supplementary materials. While I acknowledge that other approaches, such as the Kullback–Leibler divergence (Kullback and Leibler, 1951), could be employed, I ultimately opted for cosine similarity. In my view, cosine similarity offers a straightforward, well-established procedure for quantifying the angle between normalized vectors (Salton and McGill, 1983, Chowdhury, 2010), which makes it particularly intuitive for characterizing similarities in my dataset. Its ease of interpretation, combined with its broad acceptance in related empirical studies, justifies this methodological choice over other metrics.

In the context of bioethics, you might use these matrices to determine which concepts in a vignette are closely related. For example, high similarity between “treatment” and “consent” could indicate a meaningful semantic relationship relevant to informed consent discussions. The clusters of high similarity values in the matrix can help identify key themes or relationships in the vignette. If several words have high similarities to each other, it suggests a cluster of related concepts. Through the comparison of similarity matrices across various versions of a vignette (such as HC vs. LC), you can observe how word relationships alter with varying levels of detail. For instance, terms pertaining to “risks” and “treatment” may exhibit heightened resemblance in a more comprehensive version, underscoring the significance of furnishing more precise details. The matrices can help ethical analysts understand whether the language used in different scenarios (e.g., medical consent vs. decision making) affects how related concepts (like “patient,” “risk,” and “treatment”) are perceived. It could also be useful for analysing whether certain terms are ambiguous or interpreted differently in different contexts, which is essential for clarity in bioethical communication.

## Visual Analysis Techniques

The application of visual analysis techniques plays a fundamental role in understanding the relationships between concepts, especially within bioethical discourse (Sleigh et al. 2020). Visualizing the semantic relationships among terms or words can highlight subtle differences, underlying themes, or emergent patterns that are less obvious in raw numerical matrices. This section focuses on the different visual techniques used to analyse the structured matrices generated from our word embeddings, starting with hierarchical clustering dendrograms.

### Hierarchical Clustering Dendrograms

Hierarchical clustering dendrograms are powerful tools for revealing relationships between words and identifying groupings of terms based on semantic similarity (Divjak and Fieller 2014). A dendrogram is an illustration of the hierarchical relationship between items. It is typically produced as a hierarchical clustering result. Dendrograms are mostly used to determine the optimal approach to group items into clusters. In this study, dendrograms were used to visualize the hierarchical structure among the tokens extracted from each version of the bioethical vignettes. By clustering similar words together, the dendrograms offer an intuitive way to examine how key concepts are associated and how these relationships differ across different vignette versions.

**Fig. 1:**
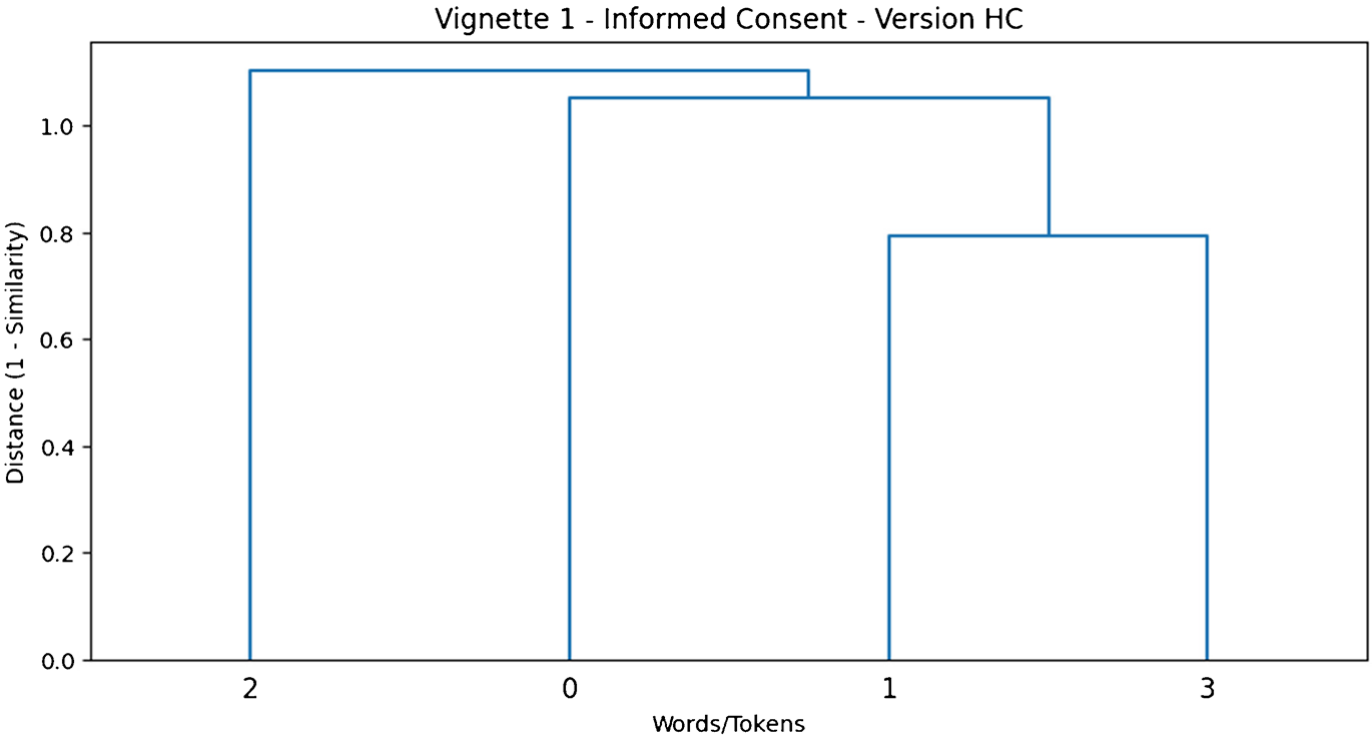
Hierarchical clustering dendrogram for the High Colexification (HC) version of Vignette 1 (“Informed Consent in Medical Procedures”). The dendrogram illustrates broader and less cohesive clusters, reflecting greater semantic ambiguity and diffuse relationships among key terms such as “treatment”, “improve”, “condition”, and “options”

**Fig. 2:**
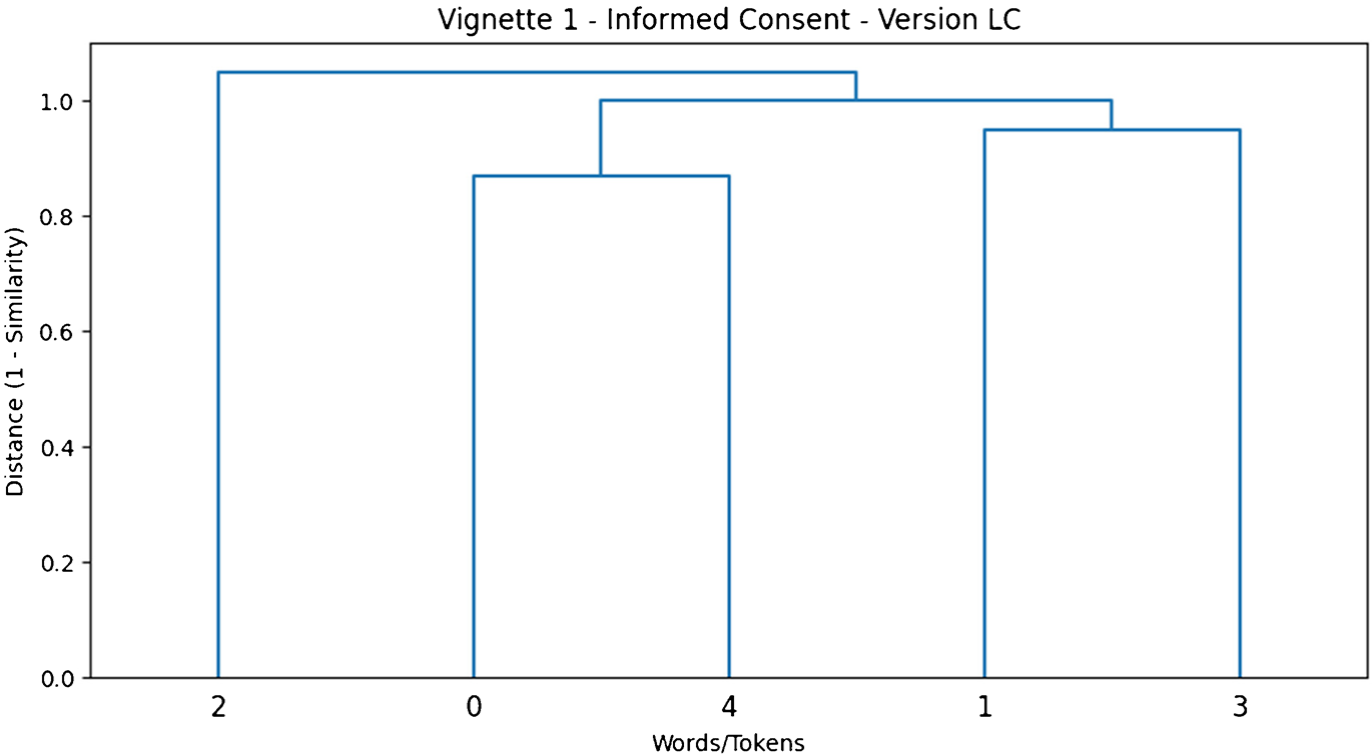
Hierarchical clustering dendrogram for the Low Colexification (LC) version of Vignette 1 (“Informed Consent in Medical Procedures”). The dendrogram demonstrates tighter and more coherent groupings, indicating reduced ambiguity and stronger semantic adjacency among specific terms such as “surgery”, “replacement”, “hip”, “osteoarthritis”, “risks”, “benefits”, “infection,” “clots”, and “recovery”

The dendrograms were constructed by applying agglomerative hierarchical clustering on the cosine similarity matrices generated from the word embeddings. Specifically, we used the average linkage method to measure the distances between clusters, which allowed for an effective representation of the overall semantic structure. The linkage method works by computing the average distance between all pairs of observations across clusters, thus capturing a balanced understanding of similarity. For each vignette, two versions (high colexification and low colexification) were analysed to observe the effects of varying levels of semantic complexity on word clustering. The hierarchical clustering approach allowed us to determine which words or phrases tended to cluster closely and whether these groupings were consistent or diverged across different versions of the vignette. For example, in the “Informed Consent in Medical Procedures” vignette, the terms “treatment” “risks” and “consent” clustered closely in the high colexification version, reflecting a strong thematic connection among these elements. In contrast, the low colexification version showed a looser connection, with clusters focusing more on isolated concepts. The dendrograms also allowed for a visual comparison between abstract and detailed versions of each vignette. In general, the more abstract versions (high colexification) showed broader, less defined clusters, indicating weaker associations between concepts. In contrast, the more detailed versions (low colexification) exhibited tighter, well-defined clusters, suggesting that providing more specific information strengthens semantic connections between terms. This clustering analysis is particularly valuable in bioethical discourse as it visually highlights which concepts are tightly connected, which are ambiguous, and how added detail impacts understanding. Such insights can be critical for ethical decision-making processes, where the clarity of concept relationships plays a key role in determining the outcomes of deliberation. By utilizing hierarchical clustering dendrograms, we were able to provide a visual representation that underscores the importance of detail and precision in bioethical communication. Below is the visualization in the form of dendrograms of some of the vignettes and versions. The dendrograms presented in Figs. 1 and 2 provide a comparative visualization of semantic relationships in the High Colexification (HC) and Low Colexification (LC) versions of Vignette 1, respectively. In Fig. 1, the HC dendrogram reveals broader clusters with greater distances between terms, highlighting the impact of ambiguous language on conceptual diffusion. Conversely, Fig. 2 depicts the LC dendrogram with more compact clusters, demonstrating how precise terminology enhances semantic coherence and reduces interpretative variability, thereby supporting clearer ethical deliberation in bioethical discourse.

The dendrograms (see, online supplementary material) effectively illustrate how different terms cluster based on their semantic similarity. The relationships are derived from the cosine similarity matrices computed for each vignette, which quantify how closely related the concepts are in semantic space. The dendrograms help visually represent the degree of colexification—i.e., how tightly words are grouped together based on shared meaning or context. For each vignette, two dendrograms are presented: one for the high colexification version and another for the low colexification version. These visualizations provide comparative insights. In HC versions, the clusters are generally broader and less cohesive, indicating that the language used is less specific, leading to weaker or more diffuse relationships among words or terms. In LC versions, the clustering shows tighter and more coherent groupings. This suggests that the language is more interconnected, reflecting richer semantic content and stronger associations among concepts.

## Lexical Clarity and Semantic Adjacency Principle

The concept of lexical clarity and the semantic adjacency principle are central to understanding the dynamics (and geometry) of language use in bioethical discourse, especially in contexts that involve making informed and ethically significant decisions. Lexical clarity refers to the extent to which terms used in communication are unambiguous, precise, and convey consistent meanings. The semantic adjacency principle, in contrast, speaks to how closely related words and concepts are within the linguistic structure of discourse, as represented by their positions in an embedding space. In this section, we explore how these principles influence the comprehensibility and ethical implications of the vignettes analysed. Lexical clarity was assessed by examining the differences between high and low colexification versions of each vignette. The high colexification versions were designed to provide a more detailed, explicit depiction of the scenario, with precise language and a clear articulation of key terms and their interrelationships. For example, in the Informed Consent in Medical Procedures vignette, the low colexification version included specific terms such as “hip replacement surgery,” “risks” and “recovery time,” which helped clarify what Ana was being asked to consent to. In contrast, the high colexification version used more general terms like “treatment” and “options,” which left more room for interpretation and, potentially, misunderstanding. The difference in lexical clarity has significant implications for ethical decision-making. In scenarios where individuals must make informed choices—such as consenting to medical procedures, deciding about end-of-life care, or considering organ donation—the clarity of the language used is crucial. Without precise language, individuals may struggle to understand the full scope of their choices, potentially leading to uninformed or less autonomous decisions. The semantic adjacency principle refers to the clustering of words in the semantic space as represented in the cosine similarity matrices and visualized in the hierarchical clustering dendrograms. Words that frequently occur together in similar contexts will have vectors that are close to each other in the embedding space, resulting in high cosine similarity values. The dendrograms generated for each vignette illustrate how terms cluster differently based on the level of detail provided in the vignette. In the detailed versions, key terms such as “treatment,” “consent,” and “risks” tended to form tighter clusters, indicating a high degree of semantic adjacency. This suggests that these terms are conceptually linked in a way that is both meaningful and helpful for understanding the situation at hand. For instance, in the Use of Experimental Therapies vignette, the clustering of terms like “clinical trial,” “gene therapy,” and “risks” reflects a coherent grouping that aids in grasping the potential consequences of Miguel’s decision. This clustering not only facilitates comprehension but also ensures that the ethical implications are framed clearly, helping individuals to make more nuanced decisions. In contrast, the abstract versions of the vignettes showed more dispersed clusters, with lower cosine similarity between key terms. This dispersion indicates weaker semantic adjacency, where terms such as “treatment” and “decision” are less directly connected in the discourse. This lack of tight clustering suggests that participants may need to work harder to make the connections between concepts, which can increase cognitive load (Sweller 1988) and hinder effective decision-making (Myser et al. 1995). The interplay between lexical clarity and semantic adjacency has important implications for bioethical communication. High lexical clarity and strong semantic adjacency facilitate a more informed understanding of ethical issues, as participants can more easily grasp the relationships between key concepts. This relationship is evident in the high colexification versions of the vignettes, where clearer language led to stronger clustering and, presumably, better comprehension. Conversely, lower lexical clarity, as seen in the abstract versions, resulted in weaker clustering of terms and a higher likelihood of misinterpretation. For ethical decision-making, this can mean the difference between an informed consent process that respects autonomy and one that leaves participants unsure about the nature of the decision they are making. We can enhance the communication of complicated bioethical concerns and empower people to make more informed and morally sound judgements by implementing the concepts of lexical clarity and semantic adjacency. The results presented in this part indicate that the quality of bioethical discourse can be greatly improved by carefully choosing language that meets the cognitive and ethical needs of decision-makers while also guaranteeing high clarity and strong semantic linkages.

## Discussion: Bioethical Analysis and LLMs

The integration of LLMs into bioethical analysis introduces both promising opportunities and significant challenges, particularly in relation to concepts such as lexical clarity and semantic adjacency. In this section, we will explore the role LLMs can play in facilitating or hindering ethical deliberation, drawing on both empirical evidence from our structured matrices and insights from recent philosophical discussions on LLM capabilities (Millière and Buckner 2024a, 2024b). LLMs, such as GPT-3 and beyond, Gemini, Claude, etc., have demonstrated an ability to generate coherent and contextually relevant text. However, the degree of lexical clarity they achieve is highly dependent on the prompt quality and the specific model’s training data. As Millière and Buckner (2024a) argue, the distinction between a model’s performance in generating seemingly linguistic responses and its true competence in understanding those language principles remains a critical concern. While LLMs can often produce lexically clear text, this clarity does not necessarily mean the model comprehends the ethical ramifications of its output (Rao et al. 2023). In the analysis of the bioethical vignettes, I observed that lexical clarity significantly influences or at least a priori, ethical decision-making. High lexical clarity, exemplified by specific and detailed terms, leads to better comprehension and more informed decisions. However, when LLMs are involved in generating these vignettes or augmenting ethical discussions, the lexical clarity may fluctuate based on the nuances of language use that the model might not fully grasp. As Millière and Buckner (2024a) note, the reliance on LLMs for semantically significant contexts may pose risks due to the model’s inability to truly understand or interpret complex semantic nuances, despite generating linguistically sophisticated content. However, despite the iterations and constant prompting, I have used OpenAI and Gemini models for the reanalysis of the vignettes and relied on them for the visualization of the illustrations and the revised result is identical to the one I had obtained without their assistant. The semantic adjacency principle explored in this study provides a framework for evaluating how LLMs might help visualize and understand relationships between ethical concepts. I illustrated how linguistic detail affects the clustering of important words or terms (even phrases) and, in turn, the semantic linkages that serve as the basis for ethical discussion by using structured matrices and creating hierarchical clustering dendrograms. The limitations discussed by Millière and Buckner, particularly around benchmarking saturation and construct validity (2024a), emphasize the importance of caution when deploying LLMs in ethically sensitive areas. Benchmarks used to evaluate LLMs often fail to capture the nuance required for true ethical competence. This is particularly pertinent when considering how LLMs might assist in ethical analysis of bioethical scenarios. For instance, while LLMs may accurately replicate the language used in detailed, high-colexification versions of the vignettes, their ability to truly grasp the ethical significance of concepts like “autonomy” and “informed consent” is inherently limited. Despite these limitations, LLMs hold potential as supportive tools for ethical analysis rather than as independent ethical decision-makers. By using LLMs to generate first drafts of ethical deliberations, followed by rigorous human evaluation, it is possible to combine the efficiency and language capabilities of LLMs with the nuanced understanding that human ethicists bring to the table. The pragmatic utility of LLMs lies not in their capacity to make decisions autonomously but in their ability to highlight potential ethical dilemmas, frame questions, and provide linguistic clarity in complex discussions such bioethical discourse. Building on the strengths and acknowledging the limitations of LLMs, it is possible to envision innovative roles for these models beyond text generation or assistance in the analysis of bioethical discourse, positioning them as ethical simulators. LLMs could be used to simulate multiple ethical viewpoints, providing a sandbox for ethicists to explore the consequences of different ethical frameworks in real time. For example, an LLM could be prompted to generate arguments for and against specific bioethical decisions, such as the use of experimental therapies or organ donation in high-stakes medical contexts. Millière and Buckner (2024b) argue that LLMs, while lacking genuine semantic competence, could serve as powerful tools for expanding the scope of ethical deliberation. We can take use of LLMs’ capacity to comprehend and produce language from a variety of ethical theories and viewpoints by framing them as instruments for ethical inquiry as opposed to final sources of ethical authority. This could enhance the conversation by pointing out potential ethical blind spots and offering a variety of viewpoints that would not come up in conventional ethical deliberation methods. In addition, LLMs could serve as a basis for educational tools in bioethics, helping students and professionals alike to engage with complex scenarios by offering a variety of ethical lenses through which to examine a case. Users could improve their comprehension of the complexities involved in bioethical decision-making by engaging with LLM-generated material that gives competing opinions. Such an application would be consistent with a constructivist approach to ethics teaching, in which students actively interact with many points of view in order to develop a sophisticated comprehension of ethical concepts. The potential for human-AI collaboration in bioethics is one of the most promising avenues for LLM application (Verma et al. 2023). Rather than viewing LLMs as standalone tools for ethical decision-making, they can be integrated into a collaborative workflow where human experts guide and refine the outputs generated by these models. As demonstrated in this analysis, LLMs are capable of mapping out the semantic relationships between concepts, which can be invaluable in highlighting areas of ethical ambiguity or tension (He et al. 2023; Stallinga et al. 2015). However, it is essential that human oversight remains central to this process. The ethical frameworks applied by LLMs are, by nature, shaped by the data they are trained on (Bender, et al. 2021), which can reflect biases and gaps inherent in the training corpus (Floridi 2020). Human experts must therefore act as ethical moderators, ensuring that the output generated by LLMs aligns with the ethical standards and cultural sensitivities appropriate to the context at hand. To fully explore the role of LLMs in ethical deliberation, it is crucial to examine how these models interact with specific ethical frameworks such as deontology or utilitarianism (Sorin et al. 2024). For instance, deontological ethics focuses on duties and rules, while utilitarianism emphasizes outcomes and maximizing overall good. LLMs, due to their nature, lack intrinsic moral understanding but could be trained to identify and prioritize actions that align with these frameworks based on extensive textual analysis of ethical codes. A critical challenge in integrating LLMs into ethical deliberation is the black box problem—the difficulty in understanding the internal decision-making processes of these models (Pasquale 2015). As LLMs grow more complex, it becomes increasingly challenging to explain why a particular output was generated. This opaqueness is particularly problematic in ethical contexts where transparency is key to ensuring trust and accountability. Millière and Buckner (2024b) discuss the importance of mechanistic interpretability as a potential avenue for addressing the black box problem. In the context of bioethics, ensuring that the reasoning behind an LLM’s ethical judgment is transparent can help mitigate concerns about reliability and trustworthiness. This might involve developing new tools for visualizing the decision pathways within LLMs or employing methods that allow for interrogation of model outputs. Such transparency is not only necessary for fostering trust but also for ensuring that the outputs of LLMs align with ethical standards and do not inadvertently cause harm. As I mentioned above, LLMs are trained on large datasets that often contain inherent biases, which can be inadvertently learned and amplified by the model. This presents a significant challenge for the ethical use of LLMs, particularly in bioethical contexts where fairness and non-discrimination are crucial. If left unchecked, these biases could lead to biased ethical judgments that disproportionately affect certain groups, thereby perpetuating social inequalities (Eubanks 2018). To mitigate this risk, it is imperative to use diverse and representative training data and to apply rigorous evaluation processes that identify and correct biased outputs. Strategies such as counterfactual data augmentation—where the training data is artificially modified to ensure fairness—can help reduce bias in LLM outputs. Furthermore, ongoing monitoring and iterative retraining of models are necessary to ensure that LLMs remain fair and unbiased over time, especially as they are applied to new bioethical scenarios. The increasing reliance on LLMs for ethical guidance raises concerns about moral deskilling—a phenomenon where individuals lose their ability to engage in moral reasoning due to over-reliance on automated systems (Vallor 2015). If ethicists and healthcare professionals become too dependent on LLMs, there is a risk that their own capacity for moral judgment could atrophy over time, leading to a diminished ability to navigate complex ethical situations independently. Addressing this issue requires a balanced approach that encourages active human engagement in ethical deliberation. LLMs should be used as tools to facilitate discussion, rather than replace human reasoning. We can support and even improve users’ moral reasoning abilities by framing LLMs as assistive tools that offer a range of viewpoints as opposed to final solutions. Instead of letting human abilities decline, we may create an atmosphere where human and machine intelligence complement each other by encouraging professionals to critically assess and improve the outputs of LLMs. Ethical decision-making often involves more than just logical analysis; it requires emotional intelligence and intuition (Hauser 2006). LLMs, however, lack the capacity for genuine emotional response or empathy, which limits their ability to fully engage with the emotional dimensions of ethical dilemmas. This limitation is particularly evident in bioethical scenarios that involve deeply personal decisions, such as end-of-life care or reproductive choices. Emotional factors are essential to understanding patient values, family dynamics, and the human elements that define ethical decision-making. While LLMs can analyse textual data and infer sentiment based on word associations, they cannot experience or genuinely empathize with the emotional weight carried by individuals facing ethical decisions. This gap presents a significant limitation in using LLMs as sole agents of ethical guidance. Nevertheless, they can still be useful by providing preliminary analyses that human decision-makers can supplement with their emotional and intuitive understanding.

## Future Directions

The application of computational analysis in bioethical contexts reveals promising avenues for future research. The study of natural dialogues in clinical settings, such as patient–doctor consultations and ethical committee deliberations, offers a fundamental pathway to validate linguistic analysis methodologies. Empirical evidence derived from these contexts can illuminate the relationship between linguistic clarity and ethical decision-making (Zwart 2021). The integration of multimodal data represents a significant advancement in this field. Recent advancements in computational multimodal analysis have significantly enhanced our ability to integrate nonverbal elements, thereby enriching the understanding of how ambiguity affects ethical deliberation. For instance, Petković et al. (2025) introduced a novel computational model that analyses nonverbal social behaviour by integrating multimodal cues such as facial expressions, gesture intensity, and spatial dynamics. Similarly, Zhou et al. (2024) developed methods to detect nonverbal behaviours, including speech and gaze, using multimodal data and computer vision techniques, which can be instrumental in interpreting collaborative interactions. All these studies using different multimodal techniques to analyse communication can be complemented by the use of LLMs in a bioethical context to provide practitioners with elements to determine the clarity of bioethical discourse. In this study I have conducted the vignettes were crafted based on themes commonly encountered in bioethics, ensuring relevance while allowing for controlled variation in linguistic precision. Future studies will analyse naturally occurring cases to validate these findings. To my knowledge, no existing studies or repositories within platforms like Kaggle, UCI Machine Learning Repository, or Google Dataset Search include specific corpora of bioethical interactions that would allow for the application of LLMs to analyse bioethical discourse comprehensively. However, future research could explore the creation of specialized bioethical vignette batteries derived from textual corpora of real-world interactions. This approach would provide a robust foundation for leveraging LLMs to uncover insights into the nuances of ethical communication in healthcare and other contexts. This approach would allow researchers to systematically apply LLM-based methods to analyse the nuances of bioethical discourse in real-world settings.

## Concluding remarks

In this article, I have explored the potential role of LLMs in bioethical analysis, highlighting both their strengths and limitations. The structured matrix approach and subsequent semantic analysis provided a unique lens through which to evaluate the capacity of LLMs to facilitate ethical deliberation. The findings reveal that while LLMs are capable of generating lexically clear and contextually relevant content, they lack the depth of ethical understanding required for autonomous decision-making. The importance of lexical clarity and semantic adjacency in ethical contexts cannot be overstated, as these elements fundamentally shape the quality and comprehensibility of ethical discourse. Furthermore, issues such as the black box problem, bias amplification, and moral deskilling underscore the need for careful human oversight and critical evaluation when integrating LLMs into bioethical workflows. Future research should focus on enhancing the interpretability of LLMs, improving mechanisms for human-AI collaboration, and developing educational tools that leverage LLMs to enrich ethical understanding without compromising human moral agency. We can use LLMs to help with ethical analysis while maintaining human oversight by framing them as helpful tools rather than as autonomous ethical decision-makers. The most hopeful way forward is to take this approach, which will enable LLMs or other AI techniques to make a significant contribution to the developing field of bioethics without compromising the fundamentally human aspects of moral reasoning as in fact its use can be of broad benefit to other areas of scientific research (Gao and Wang 2024).

## Supplementary Information

Below is the link to the electronic supplementary material.

Supplementary file1 (DOCX 14 KB)

## Data Availability

For further details, please refer to the dataset available in the supplemental material

## Footnotes

1 In this study, “ethical discourse” refers specifically to the language used in constructing the bioethical vignettes, whereas “ethical deliberation” pertains to the process of interpreting and resolving these ethical scenarios. But if one were to apply this technique to a textual corpus of bioethical interactions (e.g. doctor–patient relationships) based on transcripts or cases presented in ethical or hospital committees, it would refer to the language used to present or describe the case.

2 Although human intuition can identify examples of ambiguous versus precise language, LLMs systematically quantify these patterns, providing a scalable tool for large datasets. Unlike other disciplines, bioethics directly impacts personal autonomy and moral agency. Linguistic clarity in bioethical discourse is essential to respect these principles, particularly in high-stakes contexts such as end-of-life care and informed consent.

3 I acknowledge that the premise—namely, that ethical discourse is enhanced by increased lexical clarity—is contentious and not universally accepted. Indeed, one reviewer of this paper disagrees with it. Nevertheless, in bioethical contexts, terminological precision can facilitate more accurate communication among stakeholders, especially in life-or-death scenarios such as end-of-life decision-making or the allocation of scarce medical resources.

4 The use of vignettes allows for controlled manipulation of linguistic variables, ensuring a focus on semantic clarity without the confounding factors present in interactional texts, such as dialogic turn- taking and extralinguistic cues. See, “Future directions” section for a potential application of this technique in a more ecologically valid interactional textual corpus.

5 The vignettes were crafted based on themes commonly encountered in bioethics, ensuring relevance while allowing for controlled variation in linguistic precision. Future studies, see section on Future directions, will analyse naturally occurring cases to validate these findings. Additional information is available in the supplemental material.

## Availability of Data and Materials

All data and materials used in this study are available at https://figshare.com/account/home#/projects/223782
