# Supplementary material for: The Geometry of Language: Understanding LLMs in Bioethics
Source: J Bioeth Inq. 2025 Sep 11;22(3):573–86. doi: 10.1007/s11673-025-10480-1 (PMC12575457; doi:10.1007/s11673-025-10480-1)
Supplement: Supplementary file 1 — Supplementary file1 (DOCX 14 KB) [file 11673_2025_10480_MOESM1_ESM.docx]

Astobiza, A.M. 2025. The geometry of language: Understanding LLMs in bioethics. *Journal of Bioethical Inquiry* 22(3).

**Supplementary Material**

# Code to Generate Hierarchical Clustering Dendrograms for Vignette Matrices

import numpy as np

import scipy.cluster.hierarchy as sch

import matplotlib.pyplot as plt

# Example cosine similarity matrices for both versions of the six vignettes

vignettes_matrices = {

"Vignette 1 - Informed Consent": {

"Version HC": [

[1.00, 0.25, 0.15, 0.30],

[0.25, 1.00, 0.20, 0.45],

[0.15, 0.20, 1.00, 0.35],

[0.30, 0.45, 0.35, 1.00]

],

"Version LC": [

[1.00, 0.32, 0.18, 0.28, 0.40],

[0.32, 1.00, 0.27, 0.33, 0.29],

[0.18, 0.27, 1.00, 0.25, 0.37],

[0.28, 0.33, 0.25, 1.00, 0.31],

[0.40, 0.29, 0.37, 0.31, 1.00]

]

},

"Vignette 2 - End-of-Life Decisions": {

"Version HC": [

[1.00, 0.29, 0.22],

[0.29, 1.00, 0.33],

[0.22, 0.33, 1.00]

],

"Version LC": [

[1.00, 0.41, 0.24, 0.35, 0.39],

[0.41, 1.00, 0.38, 0.40, 0.36],

[0.24, 0.38, 1.00, 0.29, 0.34],

[0.35, 0.40, 0.29, 1.00, 0.42],

[0.39, 0.36, 0.34, 0.42, 1.00]

]

},

"Vignette 3 - Organ Donation": {

"Version HC": [

[1.00, 0.30, 0.19],

[0.30, 1.00, 0.28],

[0.19, 0.28, 1.00]

],

"Version LC": [

[1.00, 0.35, 0.31, 0.40],

[0.35, 1.00, 0.36, 0.37],

[0.31, 0.36, 1.00, 0.33],

[0.40, 0.37, 0.33, 1.00]

]

},

"Vignette 4 - Assisted Reproduction": {

"Version HC": [

[1.00, 0.28, 0.32],

[0.28, 1.00, 0.24],

[0.32, 0.24, 1.00]

],

"Version LC": [

[1.00, 0.39, 0.27, 0.31, 0.33],

[0.39, 1.00, 0.34, 0.42, 0.36],

[0.27, 0.34, 1.00, 0.38, 0.29],

[0.31, 0.42, 0.38, 1.00, 0.41],

[0.33, 0.36, 0.29, 0.41, 1.00]

]

},

"Vignette 5 - Use of Experimental Therapies": {

"Version HC": [

[1.00, 0.33, 0.25],

[0.33, 1.00, 0.31],

[0.25, 0.31, 1.00]

],

"Version LC": [

[1.00, 0.42, 0.26, 0.39, 0.30],

[0.42, 1.00, 0.37, 0.34, 0.41],

[0.26, 0.37, 1.00, 0.29, 0.33],

[0.39, 0.34, 0.29, 1.00, 0.38],

[0.30, 0.41, 0.33, 0.38, 1.00]

]

},

"Vignette 6 - Prenatal Diagnosis and Reproductive Decisions": {

"Version HC": [

[1.00, 0.27, 0.23],

[0.27, 1.00, 0.30],

[0.23, 0.30, 1.00]

],

"Version LC": [

[1.00, 0.40, 0.31, 0.36],

[0.40, 1.00, 0.39, 0.42],

[0.31, 0.39, 1.00, 0.33],

[0.36, 0.42, 0.33, 1.00]

]

}

}

# Function to generate dendrogram for a given matrix

def generate_dendrogram(matrix, title):

# Convert cosine similarity to distance (1 - similarity)

distance_matrix = 1 - np.array(matrix)

# Perform hierarchical clustering

linkage_matrix = sch.linkage(distance_matrix, method='average')

# Plot the dendrogram

plt.figure(figsize=(10, 5))

sch.dendrogram(linkage_matrix, orientation='top', distance_sort='ascending')

plt.title(title)

plt.xlabel('Words/Tokens')

plt.ylabel('Distance (1 - Similarity)')

plt.show()

# Generate dendrograms for all vignettes and their versions

for vignette, versions in vignettes_matrices.items():

for version_name, matrix in versions.items():

title = f"{vignette} - {version_name}"

generate_dendrogram(matrix, title)
